# A Retrospective Cohort Study of Leptospirosis in Crete, Greece

**DOI:** 10.3390/tropicalmed10080209

**Published:** 2025-07-25

**Authors:** Petros Ioannou, Maria Pendondgis, Eleni Kampanieri, Stergos Koukias, Maria Gorgomyti, Kyriaki Tryfinopoulou, Diamantis Kofteridis

**Affiliations:** 1 School of Medicine, University of Crete, 71003 Heraklion, Greece; 2 Internal Medicine Department, University Hospital of Heraklion, 71110 Heraklion, Greece; 3 Internal Medicine Department, General Hospital of Chania, 73300 Chania, Greece; 4 Internal Medicine Department, Venizeleio General Hospital, 71409 Heraklion, Greece; 5 Clinical Microbiology Department, University Hospital of Heraklion, 71110 Heraklion, Greece

**Keywords:** leptospirosis, weil disease, zoonosis

## Abstract

Introduction: Leptospirosis is an under-recognized zoonosis that affects both tropical and temperate regions. While it is often associated with exposure to contaminated water or infected animals, its presentation and epidemiology in Mediterranean countries remain incompletely understood. This retrospective cohort study investigates the clinical and epidemiological profile of leptospirosis in Crete, Greece, a region where data are scarce. Methods: All adult patients with laboratory-confirmed leptospirosis admitted to three major public hospitals in Crete, Greece, between January 2019 and December 2023 were included in the analysis. Diagnosis was made through serologic testing along with compatible clinical symptoms. Results: A total of 17 patients were included. Their median age was 48 years, with a predominance of males (70.6%). Notably, more than half of the patients had no documented exposure to classic risk factors such as rodents or standing water. Clinical presentations were varied but commonly included fever, fatigue, acute kidney injury, and jaundice. Of the patients who underwent imaging, most showed hepatomegaly. The median delay from symptom onset to diagnosis was 11 days, underscoring the diagnostic challenge in non-endemic areas. Ceftriaxone was the most frequently administered antibiotic (76.5%), often in combination with tetracyclines or quinolones. Despite treatment, three patients (17.6%) died, all presenting with severe manifestations such as ARDS, liver failure, or shock. A concerning increase in cases was noted in 2023. Conclusions: Leptospirosis can present with severe and potentially fatal outcomes even in previously healthy individuals and in regions not traditionally considered endemic. The relatively high mortality and disease frequency noted emphasize the importance of maintaining a high index of suspicion. Timely diagnosis and appropriate antimicrobial therapy are essential to improving patient outcomes. Additionally, the need for enhanced public health awareness, diagnostic capacity, and possibly environmental surveillance to control this neglected but impactful disease better, should be emphasized.

## 1. Introduction

Leptospirosis is a neglected, zoonotic, waterborne tropical disease caused by spirochetes belonging to the genus *Leptospira* [1]. Leptospirosis is considered a major emerging and re-emerging infectious disease by the World Health Organization (WHO), and it is widely spread among animals and humans globally [1,2]. This disease has been known by various names worldwide, including Weil’s disease, Weil-Vasiliev disease, Swineherd’s disease, nanukayami fever, cane-cutter fever, swamp fever, mud fever, Fort Bragg fever, Stuttgart disease, and Mgunda fever [3,4,5,6,7].

*Leptospira* are highly motile, spiral-shaped, aerobic spirochetes that have 18 or more coils per bacterial cell. Since the pathogens do not stain adequately with common laboratory stains, they are best visualized using dark-field microscopy, fluorescent microscopy, or silver staining. It can be differentiated from the other spirochetes based on its characteristics, with a “question mark” hook visible at the end of its bacterial cell [8].

Several changes in the phylogeny of *Leptospira* have been made since 2018, primarily due to whole-genome sequencing analysis. Currently, there are at least 64 recognized species divided into two clades and four subclades [9,10]. The subclade P1 contains 17 species, including those traditionally recognized as pathogenic, such as *Leptospira interrogans*. The subclade P2 contains 20 species, including those that were previously regarded as of intermediate or unclear pathogenicity. The subclades S1 and S2 include 22 and 5 species, respectively, and include those species previously categorized as ‘saprophytes’. An older classification system that is based on serology includes approximately 300 serovars of pathogenic *Leptospira*, organized into 32 serogroups; however, some serovars are found in more than one *Leptospira* species. Thus, isolates are identified by both species and serovar. Additionally, molecular methods can be used to classify *Leptospira* strains beyond species [8,11,12].

Leptospirosis is thought to be the most widespread zoonosis globally, and its incidence is hard to assess reliably. It is more prevalent in tropical areas but may also occur in temperate areas. Based on relatively recent studies, it is estimated that about one million cases of leptospirosis occur globally per year, leading to 60,000 deaths, while in countries of the European Union and the European Economic Area, during 2010–2021, 12,180 confirmed leptospirosis cases were reported from 23 countries; this corresponded to a mean annual notification rate of 0.24 cases per 100,000 population [13,14,15,16]. Human cases are sporadic, but outbreaks can occur after a common exposure. Animals serve as a reservoir for *Leptospira*, but the environment can also act as a reservoir if contaminated by the urine of infected animals. More than 150 mammals have been described as natural carriers of pathogenic *Leptospira* species. The pathogen is known to live in the renal tubules of infected mammals and is released in their urine [8]. The most important animals known to be associated with cases of *Leptospira* infection in humans are rodents. Infection in rodents usually occurs in utero, during their birth, or infancy after contamination of their nest [8]. The rodents become asymptomatic carriers and release the microorganisms in their urine during their life, leading to water contamination [17].

Humans can become infected incidentally after being exposed to animals or the environment. The infection begins when the pathogen enters the human body through non-intact skin, mucous membranes, or the conjunctivae [8]. People at higher risk for leptospirosis include those with occupational exposure to animals, those involved in recreational activities that expose them to environmental sources, those who travel to endemic areas, and individuals with a low socioeconomic status [2,14,18,19,20]. The clinical spectrum of the disease ranges from asymptomatic or mildly symptomatic to severe, life-threatening disease [8]. Symptoms may include fever, rigors, myalgia, headache, and liver, kidney, and respiratory failure may occur in severe cases [8].

Although leptospirosis is often perceived as a tropical disease, it persists in temperate regions, including Southern Europe. In 2022, the EU/EEA reported 0.18 confirmed cases per 100,000 individuals, with France experiencing the highest notification rate (0.36/100,000) and accounting for over 30% of cases [21]. National studies in France (e.g., from 2019–2021) documented incidence rates ranging from 0.69 to 1.08 per 100,000, with seasonal peaks in late summer [22]. Northern Italy exhibited a rising trend in seroprevalence between 2002 and 2016, with positivity reaching up to 38% in 2016 [23]. Earlier passive surveillance in France over six decades indicated persistent, albeit low-level, endemicity, with occasional fluctuations driven by environmental factors [24]. Overall, EU/EEA trends from 2010–2021 show an average annual notification increase of 5%, despite a temporary decline during the COVID-19 pandemic. Cases of human leptospirosis in Greece have been reported in the past, and they include small cohorts of patients in the North and Western Greece [25,26]. Leptospirosis in Greece is a disease monitored by the European Centre for Disease Prevention and Control and the Greek National Public Health Organization. Based on the ECDC annual epidemiological report for 2022, Greece had a relatively stable reported incidence of around 0.13 cases per 100,000 people [21]. However, data from Mediterranean islands remains limited.

The aim of the present study was to examine leptospirosis in Crete—an insular setting with unique environmental and socioeconomic characteristics—to fill a key gap in regional surveillance and contribute to a more nuanced understanding of leptospirosis epidemiology in the broader Mediterranean context. Thus, all human cases of leptospirosis from 2019 to 2023 on the island of Crete, Greece are presented.

## 2. Materials and Methods

### 2.1. Study Type and Ethics Approval

This is a retrospective cohort study of adult patients with leptospirosis who were hospitalized from January 2019 to December 2023 in Crete, Greece. More specifically, data regarding all positive serum samples for leptospirosis during the timeframe of the study from the Microbiology Department of the University Hospital of Heraklion, the referral center for microbiological diagnosis of leptospirosis in Crete, Greece, were retrieved, and positive samples during the timeframe of the study from the University Hospital of Heraklion, Heraklion, the Venizeleion General Hospital, Heraklion, and the General Hospital of Chania, Chania, Greece were included. Patients’ data were then retrieved and evaluated. In-hospital mortality was assessed based on the outcome of discharge; these data were extracted from the electronic medical records of the three participating hospitals during the investigation period. The post-COVID-19 era was defined as the era from 2020 until the end of the study. We defined “recent contact” with potential leptospiral sources as exposure occurring within 30 days prior to symptom onset. This aligns with established leptospirosis incubation periods, which typically are less than 30 days across temperate and Mediterranean settings [27].

Patients’ serum specimens were examined for the presence of IgM antibodies against *Leptospira* spp. using an enzyme-linked immunoassay (EIA) dot technique, the GenBio IgM ImmunoDOT Leptospira test (GenBio, San Diego, CA, USA), according to the manufacturer’s instructions and as described earlier [28]. This test is a rapid, easy-to-use, semi-quantitative enzyme immunoassay (EIA) that specifically detects IgM antibodies against *Leptospira biflexa* (serovar Patoc I).

The study was approved by the Institutional Review Board of the University Hospital of Heraklion (protocol code 22; 13 August 2024), the Venizeleio General Hospital (protocol code: 12; 18 June 2024), and the General Hospital of Chania (7th healthcare region of Crete approval: 31506; 30 July 2024).

### 2.2. Statistics

Descriptive statistics were calculated using GraphPad Prism 6.0 (GraphPad Software, Inc., San Diego, CA, USA). Data are presented as numbers (%) for categorical variables and medians [interquartile range (IQR)] for continuous variables.

## 3. Results

### Patients’ Characteristics

In total, 17 patients were diagnosed with leptospirosis based on compatible clinical characteristics and laboratory confirmation using an enzyme-linked immunoassay (EIA) dot technique. These patients were admitted to the three larger hospitals on the island of Crete, namely, the University Hospital of Heraklion (four patients; 23.53%), the Venizeleion General Hospital (four patients; 23.53%), and the General Hospital of Chania (nine patients; 52.94%). Males accounted for 12 (70.6%) cases, and the median age of the patients was 48 years (range 17–80 years). Most patients (76.47%) had no comorbidities. Table 1 shows the characteristics of patients diagnosed with leptospirosis who were admitted to the three hospitals.

**Figure 1:**
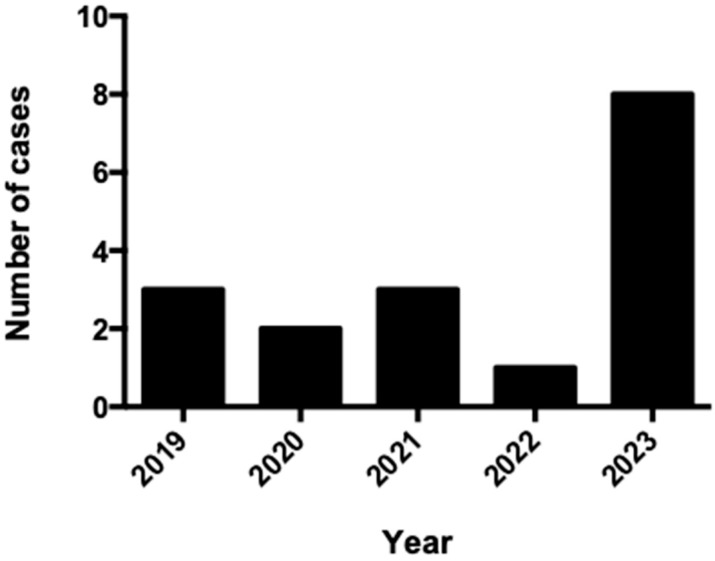
Number of leptospirosis cases per year during the study period.

Leptospirosis was diagnosed in three patients (17.6%) in 2019, in two patients (11.8%) in 2020, in three patients (17.6%) in 2021, in one patient (5.9%) in 2022, and in eight patients (47.1%) in 2023. Figure 1 shows the distribution of cases throughout the years evaluated in the present study. Most cases of leptospirosis (eight, 47.1%) were diagnosed in summer, five (29.4%) in autumn, three (17.6%) were diagnosed in spring, and one (5.9%) was diagnosed in winter. Recent contact with rodents was reported in four patients (23.5%), while recent contact with standing waters was noted in two patients (11.8%).

During the 30 days before the onset of symptoms, four (23.52%) patients reported contact with rodents, two (11.76%) reported possible exposure to contaminated water, four (23.52%) patients reported occupational exposure, and nine patients (52.9%) did not mention any possible exposure.

The median delay between initiation of symptoms and microbiological documentation of the infection was 11 days (range: 4–19 days). Regarding patients’ past medical history, the median Charlson comorbidity index was 1 (range: 0–6). Most people did not have any significant comorbidity, while only one patient had heart failure (5.9%), one patient had cerebrovascular disease (5.9%), and one patient had severe chronic kidney disease (5.9%).

Regarding clinical presentation, fever and weakness occurred in 13 (76.5%) patients, 4 (25%) patients complained of strong headache, 3 (17.6%) had diarrhea, 7 (41.2%) developed acute renal failure, 4 (25%) had jaundice, 6 (35.3%) had anemia, and 5 (29.4%) had thrombocytopenia. Weil disease was present in four (25%) patients, and one patient was diagnosed with meningitis. In terms of severity of the disease, the median Sequential Organ Failure Assessment (SOFA) score was 2 (range: 0–16).

All patients received antimicrobial treatment for leptospirosis. The most common antimicrobial used for patients’ treatment was ceftriaxone (13 patients, 76.5%), tetracycline (10, 58.8%), a quinolone (3, 17.6%), and aminopenicillin (1, 5.9%). The in-hospital and one-month mortality was 17.6% (three patients). The median duration of hospital stay was 11 days (range: 2–78 days).

Table 1 shows a comparison of patients’ characteristics regarding mortality. Even though the small number of patients precludes statistical analysis, those who died were more likely to have a higher age, previous organ failure in their medical history, and a higher Charlson comorbidity index. Those who died were also more likely to have had typical epidemiological exposure in their history; they also had a higher SOFA score as well as higher white blood cell and bilirubin counts, and were more likely to present with acute kidney failure. More specifically, the patients who died were 25, 59, and 80 years old. Two of them were admitted to the intensive care unit (ICU), and one was admitted to the medical ward. Two out of these patients had a history of recent contact with rodents. The older one had a Charlson comorbidity index of 6, while the others had scores of 0 and 2. The presenting symptoms were loss of consciousness in one patient, shock and failure of the kidneys and liver in another one, and acute respiratory distress syndrome and acute kidney failure in the last one. In all patients, there was a delay of at least 10 days between the onset of symptoms and the diagnosis of leptospirosis. All three patients were treated with ceftriaxone, and two of them were also treated with tetracycline.

## 4. Discussion

This retrospective study offers insights into the epidemiology, clinical manifestations, diagnostic challenges, treatment approaches, and outcomes of patients admitted with leptospirosis to public hospitals of Crete, Greece, over a five-year period. Notably, this is the first report of leptospirosis cases on the island of Crete, Greece. Typical predisposing factors were only seldom reported. The clinical presentation varied, but most commonly included fever and weakness. Hepatomegaly, acute kidney disease, and jaundice were common. Ceftriaxone was the most commonly used antimicrobial for the treatment of leptospirosis. Mortality was notable, with 3 out of 17 patients dying. As one of the few cohort studies of leptospirosis in this region, it contributes valuable data to the broader understanding of leptospirosis in temperate, Mediterranean settings.

Leptospirosis remains a neglected tropical disease even though it has a global distribution and carries significant morbidity and mortality. The global burden is notable, with over one million cases of the disease annually and approximately 60,000 deaths [13]. Although leptospirosis most commonly affects tropical regions, outbreaks and sporadic cases also occur in temperate regions like Southern Europe, and they imply the need for ongoing vigilance. The findings of the current study are consistent with this global trend, and they also highlight a notable increase in leptospirosis cases in 2023, which might suggest a real increase in incidence or the possibility of increased detection and reporting of the disease.

The demographic profile of the patients presented in the current cohort aligns with previous studies. Most patients were male and of working age, with a median age of 48 years. This demographic skew is commonly seen in the literature and has been attributed to occupational and environmental exposures that disproportionately affect men [14,20,29,30]. The presence of typical factors associated with leptospirosis in the present cohort is slightly lower than that in other studies [31,32]. While classic leptospirosis transmission is linked to direct contact with contaminated water or infected animals, emerging evidence suggests that indirect routes of exposure are increasingly relevant, particularly in urbanized and peri-urban settings. *Leptospira* can survive for weeks to months in moist environments, including soil, sewage, or household surfaces, allowing transmission via contaminated hands, food, or fomites [8,33]. Aerosolized droplets from rodent urine, particularly during cleaning of enclosed, poorly ventilated spaces (e.g., garages, farms), represent another potential route [33]. Urban flooding and leaking drainage systems may spread *Leptospira* to areas far from rodent nesting zones, creating hidden exposure risk even for individuals with no occupational or rural activity [34,35]. The high proportion (52.9%) of patients in our cohort without known direct exposures underscores the likely role of these indirect, environmental, and low-awareness exposure routes, reinforcing the need for public education and environmental hygiene strategies in both urban and rural areas.

The seasonal distribution of cases of leptospirosis in the present study, which peaked during the summer months, is congruent with the known epidemiology of the disease. Warmer weather and increased rainfall facilitate the survival of *Leptospira* in the environment and increase human contact with contaminated water sources [2]. Crete’s Mediterranean climate, with its warm, dry summers and mild, wet winters, likely contributes to these seasonal trends. However, only a small fraction of the present cohort reported recent contact with standing water or rodents, which suggests that less overt exposure routes may be significant, or indirect means of exposure to the pathogen may be ignored or go unnoticed.

Clinically, the presentation of leptospirosis in the present cohort was heterogeneous, consistent with the disease’s wide clinical spectrum. Fever and weakness were the most common presenting symptoms and were noted in most of the patients. Hepatomegaly and acute kidney injury were frequently noted, and this may reflect the systemic nature of the disease and the pathogen’s affinity for hepatic and renal tissues. The incidence of Weil disease in the present cohort is high and warrants attention. This severe form of leptospirosis is characterized by jaundice, renal failure, and hemorrhagic complications, and its presence underlines the potential severity of the disease even in non-endemic regions [8].

In the present study, the median duration between symptom onset and diagnosis was 11 days. This highlights a key challenge in leptospirosis management. The non-specific symptoms at presentation and the low index of clinical suspicion in non-endemic areas may contribute to this delay. Early diagnosis is of utmost importance, as timely antibiotic treatment can mitigate complications and improve outcomes [8].

Laboratory diagnosis of leptospirosis could be achieved by culture isolation of *Leptospira* spp. from a clinical specimen, demonstration of pathogenic *Leptospira* spp. by immunofluorescence, detection of genomic nucleic acid using molecular methods, and detection of specific antibody response using several serological tests [36]. However, routine practice in clinical microbiology laboratories typically includes (a) PCR testing performed during the acute phase of infection in whole blood (collected in the first week of illness) and urine (collected after the first week of illness), and (b) serological detection employed later in the disease course to detect IgM antibodies. The sensitivity of serology increases in the second week of illness. As for serologic diagnosis of leptospirosis, microscopic agglutination test (MAT) has been the reference standard; however, it can only be performed at certain reference laboratories. Nowadays, various commercial serologic screening tests are available, including IgM enzyme-linked immunosorbent assay and ImmunoDOT/DotBlot rapid diagnostic tests [37].

As a possible limitation of the present study, leptospirosis laboratory diagnosis was based solely on the detection of IgM antibodies in patients’ sera referred to the laboratory 9–14.5 days after the disease onset. According to UK Health Security Agency guidance for leptospirosis, although IgM testing for *Leptospira* may occasionally produce non-specific reactivity, patients presenting with a compatible clinical syndrome and a positive IgM result should be treated accordingly, even in the presence of negative PCR results, a practice also used in the present study [38]. Cross-reactivity of antibodies may occur with infections such as syphilis, Lyme disease, typhoid fever, or dengue. However, the clinical presentations of these diseases are typically distinct enough to minimize confusion with leptospirosis, a fact in accordance with our experience with the present cohort. As PCR testing of acute samples is now the recommended method for leptospirosis confirmatory diagnosis in clinical microbiology laboratories instead of MAT, a real-time PCR assay for the qualitative detection of the 16S rRNA gene of pathogenic *Leptospira* spp. (AmpliSens^®^ Leptospira-FRT PCR kit, Moscow, Russia) was introduced in the reference microbiology laboratory of the present study in early 2024 in the framework of updating the clinical microbiology diagnostic stewardship. However, no urine samples were referred to the laboratory for cases reported in the current study.

Regarding treatment, ceftriaxone was the most frequently prescribed antibiotic, followed by tetracycline and quinolones. This reflects current clinical practice for leptospirosis, where these antimicrobials are the recommended standard of treatment [8,39]. For example, in other studies, both in Greece and in other countries, beta-lactams and doxycycline are the first line choices for empirical treatment of leptospirosis [26,39,40]. However, all three patients who died had received ceftriaxone, with two also receiving tetracycline. This implies that the appropriate selection of antimicrobial agents may not be the only determinant of mortality, since other significant factors such as the severity of the disease and pre-existing medical conditions may also contribute to patient outcomes. Additionally, this also raises questions about whether adjunctive therapies or earlier interventions might lead to better outcomes [39]. For example, there are studies suggesting that adjunctive corticosteroid treatment might be associated with better clinical outcomes [41,42]. However, a recent systematic review identified only five studies on this topic, with four being appropriate for assessment of the role of corticosteroids, and did not reach a positive association overall between their use and positive clinical outcomes [43].

The observed mortality rate in the present study of 17.6% is concerning, and it is higher than that reported in many other settings, in which mortality rates for hospitalized patients typically range from 5% to 15% depending on disease severity and access to care, and aligns more closely with cohorts of patients admitted to the ICU, where mortality is 20–52%, particularly among patients with pulmonary hemorrhage, multiorgan dysfunction, or late presentation [13,14]. The presence of severe disease manifestations, such as ARDS, shock, and multiorgan failure, in the patients who died underlines the need for early recognition and aggressive management of this disease. Interestingly, a recent study evaluating the risk factors of inpatients with leptospirosis in eight general hospitals in Thailand identified hemoptysis, hypotension, platelet count < 100,000/µL, white blood cell count > 14,000/µL, hematocrit ≤ 30%, and jaundice as independent factors associated with mortality in a multivariate model [44]. The literature from Taiwan and Malaysia identify factors such as shock, pulmonary involvement, hemorrhage, steroid use, and diabetes as strong predictors of mortality [45]. In the present study, among the three fatal cases, two had diabetes mellitus, one had chronic kidney disease, and two exhibited pulmonary features on imaging, mirroring these global severity profiles. However, the delay between the development of leptospirosis and its diagnosis has not been associated with disease severity, as noted in a relatively recent study [46]. The median delay to diagnosis in that study was 4 days, which is much shorter compared to the median delay in the present study, which is close to the diagnostic delay noted in all patients who died.

One striking feature of the present cohort is the relatively low Charlson Comorbidity Index (CCI), with most patients being previously healthy. This suggests that even otherwise healthy individuals are at significant risk of severe outcomes, possibly due to virulent *Leptospira* strains or host immune responses. One of the patients who died had a high CCI of 6. This underlines how comorbid conditions can exacerbate disease severity, as is also noted in the literature [14].

In comparison, studies from other regions of Greece have revealed varying clinical presentations and outcomes. For instance, Papa and Kotrotsiou and Gkentzi et al. reported on cases in Northern and Western Greece, respectively, identifying similar risk factors and clinical profiles but with generally lower mortality [25,26]. However, patients’ clinical severity may differ since not all patients in those previous studies were hospitalized. Thus, differences in clinical severity, as well as promptness of diagnosis and potential regional variations in *Leptospira* strains, may contribute to these discrepancies.

This study also reflects on the broader public health implications. The sporadic nature of leptospirosis cases in Greece in general, and Crete in particular, may lead to underdiagnosis or delayed diagnosis, especially in the absence of classic exposure history. Increasing awareness among healthcare professionals is essential. Moreover, public health authorities should consider targeted educational campaigns in high-risk populations, such as farmers, waste management workers, and individuals involved in water sports or outdoor activities. Evaluating the knowledge of people at high risk for the disease would be an important first step to allow identification of the gaps in knowledge for leptospirosis, as this would allow for better design of the educational interventions that would be required to appropriately inform those at high risk [47,48,49]. Community education campaigns in rural areas could improve knowledge of transmission, personal protective equipment such as rubber boots and gloves, and hygiene practices, such as covering cuts and avoiding floodwater. Such models have been successfully implemented in other countries, such as in Southeast Asia [50]. Increasing people’s awareness should be done in conjunction with ongoing disease surveillance from the public health authorities that would allow timely identification of an outbreak of the disease which would necessitate interventions to reduce the likelihood of progressing into an epidemic [51]. To that end, the use of geographic information systems and hydrometeorological data could allow identification of high-risk zones (flood plains and rodent habitats) and the development of outbreak-forecast models via climate-based early warning systems, as already demonstrated in other countries [52]. This would allow adequate environmental risk mapping and the use of early warning systems to aid in the prevention and adequate public health response for leptospirosis cases. Moreover, since this disease spans from animals to humans, a one-health approach could be used in an attempt to reduce cases and control the disease. There is a scoping review in that direction that proposes a framework for leptospirosis interventions across the human-animal-environment interfaces and sets a basis for policymakers and scientists [52]. For example, rodent control and infrastructure improvement through enhanced sanitation, waste management, and drainage in rural settlements, as well as coordination of systematic rodent control in identified hotspots, could aid in the prevention of leptospirosis. Finally, implementation of integrated human-animal health surveillance, including livestock vaccination where applicable, to detect animal reservoirs and guide intervention strategies could also aid towards the prevention of leptospirosis [53].

The Greek National Public Health Organization has released recent epidemiological data confirming the increase in leptospirosis cases in 2023 to a reported incidence higher than 0.9 per 100,000 people, mostly involving people 45–64 years old, occurring mostly in late summer and early autumn [54]. The marked increase in cases in 2023 in the present study prompts questions regarding environmental or societal changes. The ECDC provides epidemiological reports for leptospirosis cases in the European Union. The reports for 2021 and 2022 show that, after a reduction in the cases noted in 2020, the first year of the COVID-19 pandemic, there was an increase in the reported cases [21,55]. During the post-COVID-19 period, shifts in human behavior, mobility, and healthcare-seeking behavior have occurred, and this could be associated with changes in disease transmission patterns. Most importantly, environmental changes, such as increased urbanization or changes in rodent populations, can also play a role and merit further investigation. Notably, there are studies in smaller countries that suggest an increase in leptospirosis cases during the COVID-19 pandemic [56]; however, these patterns may not reflect those seen in larger countries. Importantly, the effect of climate change or floods should also be taken into account when evaluating the epidemic curves of this disease [57,58,59]. For example, the increase in cases in Greece in 2023 was mostly associated with the floods that devasted local communities in Thessaly, leading to a surge of leptospirosis cases [60]. The data shown in the present study that examine the phenomenon in Crete, where such phenomena were not noted could be partially associated with the abovementioned factors, such as a resurgence of leptospirosis cases after the end of the COVID-19 pandemic and the restriction measures that had been applied before, and an increase in outdoor human activity both for professional and recreational reasons. Finally, climate change, moving human populations, and changes in rodent populations could have also played a role in this increase.

Future research should aim to expand the sample size through prospective, multicenter collaboration across Greece. Molecular typing of *Leptospira* isolates could reveal circulating strains and their pathogenic potential. Environmental surveillance, including rodent sampling and water testing, would provide insights into reservoirs and transmission routes. Additionally, health economic analyses could quantify the burden of leptospirosis and justify resource allocation for prevention and control measures.

This study has some notable limitations that should be mentioned. First, the retrospective nature may introduce information bias and limit causal inferences. Second, the small sample size restricts statistical power and generalizability. Third, detailed environmental and occupational exposure histories were not consistently documented, limiting the ability to identify precise transmission pathways.

## 5. Conclusions

Based on the data from patients with leptospirosis who were admitted to public hospitals in Crete, Greece, the disease can present with a spectrum of clinical manifestations, often in the absence of classic risk factors. Diagnostic delays are common and could contribute to severe outcomes. Enhancing clinician awareness, improving diagnostic capacities, and conducting further epidemiologic and environmental research are essential steps to better understand and mitigate the impact of leptospirosis in this and similar settings. The findings of this study underscore the importance of maintaining a high index of suspicion for leptospirosis, even in non-endemic, temperate regions, particularly in the face of environmental and climatic changes that may alter traditional patterns of disease transmission.

## Figures and Tables

**Table 1:** Clinical characteristics and laboratory findings of patients admitted for leptospirosis in the given study period in Crete, Greece.

Characteristic	All Patients (*n* = 17)	Lived (*n* = 14)	Died (*n* = 3)
Male gender, *n* (%)	12 (70.6)	10 (71.4)	2 (66.7)
Age, years, median (IQR)	48 (34–58)	47 (37–57)	59 (25–80)
CCI, median (IQR)	1 (0–1.5)	0.5 (0–1)	2 (0–6)
Coronary artery disease, *n* (%)	1 (5.9)	1 (7.1)	0 (0)
Heart failure, *n* (%)	1 (5.9)	0 (0)	1 (33.3)
Cerebrovascular disease, *n* (%)	1 (5.9)	1 (7.1)	0 (0)
Chronic kidney disease, *n* (%)	1 (5.9)	0 (0)	1 (33.3)
Recent contact with rodents, *n* (%)	4 (23.5)	2 (14.3)	2 (66.7)
Recent contact with standing water, *n* (%)	2 (11.8)	1 (7.1)	1 (33.3)
Occupational exposure to animals or land, *n* (%)	4 (23.5)	3 (21.4)	1 (33.3)
Symptom onset to diagnosis, days, median (IQR)	11 (9–14.5)	10 (8.8–15)	11 (10–13)
SOFA score, median (IQR)	2 (0–8.5)	1.5 (0–6.8)	8 (0–16)
Symptoms and signs			
Fever, *n* (%)	13 (76.5)	11 (78.6)	2 (66.7)
Weakness, *n* (%)	13 (76.5)	10 (71.4)	3 (100)
Jaundice, *n* (%)	4 (23.5)	3 (21.4)	1 (33.3)
Acute renal failure, *n* (%)	7 (41.2)	4 (28.6)	3 (100)
Diarrhea, *n* (%)	3 (17.6)	3 (21.4)	0 (0)
Headache, *n* (%)	4 (23.5)	4 (28.6)	0 (0)
Dyspnea, *n* (%)	4 (23.5)	3 (21.4)	1 (33.3)
Hepatomegaly in imaging, *n* (%)	9 out of 10 (90)	7 out of 8 (87.5)	2 out of 2 (100)
Laboratory characteristics			
White blood cells/mm^3^, median (IQR)	14,500 (11,470–20,920)	14,500 (11,470–18,960)	24,880 (11,760–38,000)
Hemoglobin, g/dL, median (IQR)	12.4 (10.3–12.8)	12.4 (11.7–12.7)	10.3 (9–14.1)
Total bilirubin (max), mg/dL, median (IQR)	1.1 (0.45–4.2)	0.8 (0.4–4)	2.1 (1.4–15.9)
Direct bilirubin (max), mg/dL, median (IQR)	0.6 (0.2–2.7)	0.4 (0.2–2.6)	1.6 (0.7–11.8)
AST (max), mg/dL, median (IQR)	87 (73–100)	90 (62.3–201.8)	85 (75–98)
ALT (max), mg/dL, median (IQR)	90 (50–110.5)	96 (76.5–128.5)	54 (46–71)
γGT (max), mg/dL, median (IQR)	211 (142–216)	211 (158.5–218)	161.5 (142–181)
ALP (max), mg/dL, median (IQR)	137 (81.8–269.8)	126 (79.5–289.5)	148 (148–148)
Creatinine (max), mg/dL, median (IQR)	1.4 (0.8–2.9)	1.3 (0.7–2.1)	4.7 (2.3–6.2)
C-reactive protein, mg/dL, median (IQR)	24.2 (19.1–28)	24 (17.6–26.7)	29.7 (25.3–34)
Disease outcomes			
Duration of hospitalization, days, median (IQR)	11 (4–20.5)	11.5 (6.3–19.8)	4 (2–78)
In-hospital mortality, *n* (%)	3 (17.6)	NA	NA
30-day mortality, *n* (%)	3 (17.6)	NA	NA

γGT: gamma-glutamyl transferase; ALP: alkaline phosphatase; ALT: alanine aminotransferase; AST: aspartate aminotransferase; CCI: Charlson comorbidity index; IQR: interquartile range; NA: not applicable; SOFA: Sequential Organ Failure Assessment score; leptospirosis diagnosis was based on the presence of IgM antibodies against *Leptospira* spp. using an enzyme-linked immunoassay (EIA) dot technique.

## Data Availability

Data are available from the corresponding author upon reasonable request. The data are not publicly available due to privacy/ethical reasons.
